# Effect of spermidine on ameliorating spermatogenic disorders in diabetic mice via regulating glycolysis pathway

**DOI:** 10.1186/s12958-022-00890-w

**Published:** 2022-03-07

**Authors:** Jin-Yuan Wang, Duo Ma, Min Luo, Yong-Peng Tan, Ou Zhong, Ge Tian, Yong-Ting Lv, Mei-Xiang Li, Xi Chen, Zhi-Han Tang, Lin-Lin Hu, Xiao-Can Lei

**Affiliations:** 1 Clinical Anatomy & Reproductive Medicine Application Institute, Heng Yang Medical College, University of South China, Hengyang, 421001 Hunan China; 2 Postdoctoral Station for Basic Medicine, Hengyang Medical College, University of South China, Hengyang, 421001 Hunan China; 3 China Reproductive Medicine Center, The Affiliated Hospital of Youjiang Medical University for Nationalities, Baise, 533000 Guangxi China

**Keywords:** Diabetes, Spermidine, Spermatogenic dysfunction, Glycolytic pathway, Sertoli cells

## Abstract

Diabetes mellitus (DM), a high incidence metabolic disease, is related to the impairment of male spermatogenic function. Spermidine (SPM), one of the biogenic amines, was identified from human seminal plasma and believed to have multiple pharmacological functions. However, there exists little evidence that reported SPM’s effects on moderating diabetic male spermatogenic function. Thus, the objective of this study was to investigate the SPM’s protective effects on testicular spermatogenic function in streptozotocin (STZ)-induced type 1 diabetic mice. Therefore, 40 mature male C57BL/6 J mice were divided into four main groups: the control group (*n* = 10), the diabetic group (*n* = 10), the 2.5 mg/kg SPM-treated diabetic group (*n* = 10) and the 5 mg/kg SPM-treated diabetic group (*n* = 10), which was given intraperitoneally for 8 weeks. The type 1 diabetic mice model was established by a single intraperitoneal injection of STZ 120 mg/kg. The results showed that, compare to the control group, the body and testis weight, as well the number of sperm were decreased, while the rate of sperm malformation was significantly increased in STZ-induced diabetic mice. Then the testicular morphology was observed, which showed that seminiferous tubule of testis were arranged in mess, the area and diameter of which was decreased, along with downregulated anti-apoptotic factor (Bcl-2) expression, and upregulated pro-apoptotic factor (Bax) expression in the testes. Furthermore, testicular genetic expression levels of Sertoli cells (SCs) markers (WT1, GATA4 and Vimentin) detected that the pathological changes aggravated observably, such as the severity of tubule degeneration increased. Compared to the saline-treated DM mice, SPM treatment markedly improved testicular function, with an increment in the body and testis weight as well as sperm count. Pro-apoptotic factor (Bax) was down-regulated expression with the up-regulated expression of Bcl-2 and suppression of apoptosis in the testes. What’s more, expression of WT1, GATA4, Vimentin and the expressions of glycolytic rate-limiting enzyme genes (HK2, PKM2, LDHA) in diabetic testes were also upregulated by SPM supplement. The evidence derived from this study indicated that the SMP’s positive effect on moderating spermatogenic disorder in T1DM mice’s testis. This positive effect is delivered via promoting spermatogenic cell proliferation and participating in the glycolytic pathway’s activation.

## Introduction

Diabetes mellitus (DM) is a group of metabolic diseases characterized by chronic hyperglycemia due to a variety of genetic and environmental causes, which were now 366 million DM people around the world [1]. Moreover, in patients between the ages of 18 and 30, the prevalence was 6.5% higher in males than in females, especially in the one who had more reproductive needs [2]. As well, diabetes occurs due to sedentary life style and usage of advanced glycation end-products (AGE) in diets [3], which leads to multiple organ damage as nephropathy, cardiovascular and so on [4, 5]. It’s worth noting that male reproductive disorder is becoming one of the common complications of DM recently, such as hyposexuality, impotency and decreased fertility [6]. In particular, it can cause testicular spermatogenic dysfunction, which include damaged testicular normal structural, impaired structural and functional of SCs, decreased sperm number and motility as well increased abnormal sperm numbers [7, 8]. However, the molecular mechanisms underlying DM-induced male reproductive dysfunction is not clear.

Spermidine (SPM) has been found for the first time in semen, well-known as polyamines medicine that promotes health and anti-aging [9, 10]. Studies have shown that SPM possess multiple pharmacological functions including anti-inflammatory, promote autophagy, antioxidant, protecting the health of the nervous system and cardiovascular system [9]. Recently, several evidences have instructed the protective effects of SPM on the male reproductive damage induced by electromagnetic field, chemotherapy drugs [11]. In a diabetic animal model, SPM could attenuate diabetic myocardial fibrosis via inhibition of the canonical Wnt signaling pathway-mediated abnormal autophagy in male mice [12]. Diabetic hemoglobin glycation and lipid peroxidation induced could be alleviated by the antioxidant stress function of SPM intervention [13]. Besides, SPM could exert effects on decreasing bodyweight by improving glucose utilization [14]. However, there are few reports on the effect of SPM on improving diabetic spermatogenic disorders in male. Detailed investigation into the molecular mechanisms underlying SPM-mediated protective effects on male reproductive dysfunction induced by DM is required.

In this study, we aimed to clarify whether SPM could improve the structural function of SCs, and the protective effects of SPM on spermatogenic dysfunction by regulating glycolytic pathways in streptozotocin (STZ)-induced diabetic mice. Sperm parameters, the testicular protein levels of apoptosis-related factors (Bax, Bcl-2), SCs marker (WT1, GATA4, Vimentin), key enzyme genes for glycolysis (HK2, PKM2, LDHA) were detected closely. The present study may further advance our understanding of molecular mechanisms of SPM-mediated protective effects on DM-induced male reproductive dysfunction.

## Materials and methods

### Animals and experimental groups

Healthy 6-week-old 20 g male C57BL6J mice were purchased from the University of South China (Animal Permit NO: USC2020031602). All experimental procedures were approved by Laboratory Animal Welfare Ethics Committee (NO: 2021USA0628), University of South China. For the induction of type 1 diabetes (T1DM), mice were intraperitoneally injected single dose of 120 mg/kg STZ (Sigma S-0130) dissolved in 0.1 M citrate buffer at pH 4.5 [15]. We monitor the fasting blood glucose levels for 3 consecutive days after STZ injection, considering mice with fasting glucose levels ≥11.1 mM as diabetic.

After the STZ injection, we divide mice into four main groups, treating daily for 8 weeks with the following protocol. As the control group (control group, *n* = 10) and DM + saline group (model group, *n* = 10), which received orally administered physiological saline (1 mL/kg/d). DM + 2.5 mg/kg SPM (Sigma, S-2501) group (*n* = 10) [11] and DM + 5 mg/kg SPM group (*n* = 10) [12]. The SPM was dissolved in physiological saline, which was administered by intraperitoneal injection to the mice. After the last treatment, animals were anesthetized by intraperitoneal administration of 0.6 mg/kg Urethane. We collect relevant tissues for further analysis.

### Assessment of sperm number, abnormal sperm rate

The caudal epididymis was dissected and placed in 1.5 mL of saline media heated to 37 °C, and then cut up the tissue, to give motility sperm time to escape into liquid, the caudal epididymis was left undisturbed for 20 min. Sperm morphology were observed by stained with eosin-Y [16]. At the same time, we count the abnormal sperm and calculate the abnormal sperm rate. However, the evaluation of the sperm motility was not carried out impetuously with regard to avoidance of time.

### Histopathologic and the area and diameter of seminiferous tubules analyses

The left testis, epididymis were removed and fixed in 4% polyoxymethylene overnight for histological analysis. And the tissue was embedded in paraffin and cut 4 μm thickness by microtome. The slides were stained with hematoxylin and eosin (H&E) for observing the morphology. Then, the diameters and area of 50 seminiferous tubules from each group were randomly evaluated by using an ocular micrometer with a light microscope.

### Quantitative real-time polymerase chain reaction (qRT -PCR)

Total RNA from mice testicular tissues was extracted using TRIzol reagent (Invitrogen, Carlsbad, USA). mRNA was reverse converted into cDNA using the PrimeScript 1st strand cDNA Synthesis Kit (Takara Bio, Dalian, China). Real-time PCR was performed on an ABI7900 PCR system (Applied Biosystems, Foster City, USA) using SYBR Green Real-Time PCR Master Mix (ThermoFisher SCIENTIFIC NO:4309155). Endogenous GAPDH control was used as an internal control for relevant mRNA expression [17]. The relative expression of PCNA mRNA was assessed using the comparative Ct method. The sequences of the primers used are shown in Table 1.

**Table 1:** Primers sequences used as target and reference genes used in qPCR reactions

Gene	Sequence of forward and reverse primers 5′-3’	Accession no.
Bax	F:TGCAGAGGATGATTGCTGAC	NM_007527.3
	R:GATCAGCTCGGGCACTTTAG	
Bcl-2	F:GGTGGTGGAGGAACTCTTCA	NM_177410.2
	R:ATGCCGGTTCAGGTACTCAG	
LDHA	F:ACTGTGTAACTGCGAACTCC	BC094019.1
	R:GGGAATGATGAACTTGAAGA	
HK2	F:CGTGGTAAATGACACAGTTG	BC054472.1
	R:AGTTCCACATTACGCATCTC	
PKM2	F:CAGTACAGAATACACACCCA	BC094663.1
	R:GTCATGTCTTATGTGTGGGT	
GATA4	F:ATGCCTGTGGCCTCTATCAC	AF179424.1
	R:GGTGGTGGTAGTCTGGCAGT	
WT1	F:ATCCGCAACCAAGGATACAG	DQ537939.1
	R:GGTCCTCGTGTTTGAAGGAA	

### Western blot

The testis lysed in ice-cold RIPA lysis buffer (Promega corporation, Madison Wi, uSa) and centrifuged t 14,000 rpm for 10 min at 4 °C. The concentrations of total protein in the whole-cell lysate were calculated via Bradford’s procedure. An equal amount (20 μg) of protein in each sample was separated by 12% sodium dodecyl sulphate-polyacrylamide gel electrophoresis and then transferred to a polyvinylidenedifluoride (PVDF) membranes (Millipore, Billerica, MA, USA). The membranes were incubted via primitive antibodies overnight at 4 °C with horseradish peroxidase-conjugated goat anti-rabbit IgG (H + L) secondary antibody, Alexa Fluor 594 (R37117) and immersed in ECL Plus Western Blotting detection reagent and displayed on Hyperfilm ECL (both from Amersham, Piscataway, NJ, USA). The band’s intensity was determined using Lab Works 4.5 software (UVP, Upland, CA, USA) [18]. The primary antibodies used for Western blotting were tubulin, HK2, PKM2, LDHA. The information of antibody used in the experiment are shown in Table 2.

**Table 2:** antibody used as target and reference used in immunohistochemistry and Western Blot reactions

Antibody	Company	Accession no.	Concentration of antibodies in immunohistochemistry	Concentration of antibodies in Western Blot
Vimentin (D21H3) XP® Rabbit mAb	Cell Signaling Technology	#5741	1:200	
LDHA (C4B5) Rabbit mAb	Cell Signaling Technology	#3582	1:200	1:1000
PKM2 (D78A4) XP® Rabbit mAb	Cell Signaling Technology	#4053	1:500	1:1000
HK2 Rabbit pAb (A0994)	ABclonal Technology	#3099	1:200	1:1000
Bax (D3R2M) Rabbit mAb	Cell Signaling Technology	#14796	1:100	
Bcl-2 Rabbit mAb	ABclonal Technology	#19693	1:100	
SignalStain® Boost IHC Detection Reagent (HRP, Mouse)	Cell Signaling Technology	#8125	1:200	
Biotin-conjugated Affinipure Goat Anti-Rabbit IgG(H + L)	Protein Tech Group Inc., USA	SA00004-2	1:200	
HRP-conjugated Streptavidin	Protein Tech Group Inc., USA	SA00001-0	1:200	
alpha Tubulin Monoclonal Antibody (236-10,501)	Thermofisher scientific	A11126		1:3000
HRP-conjugated affinipure goat anti-mouse IgG (H + L)	Protein Tech Group Inc., USA	SA00001-1		1:5000
goat anti-rabbit IgG (H + L)	Protein Tech Group Inc., USA	SA00001-2		1:5000

### Immunohistochemical analysis of apoptosis-related factors (Bax, Bcl-2), SCs marker (Vimentin) and key enzyme genes for glycolysis (HK2, PKM2, LDHA)

The 4-μm-thick dewaxed slices were rehydrated using gradient ethanol, incubated with pH 6.0 citrate buffer with an application of high voltage for 3 min. Then, the sections were incubated with a monoclonal mouse antibody against Bax, Bcl-2, Vimentin, HK2, PKM2, LDHA primary antibodies followed by anti-rabbit IgV for 60 min at 4 °C overnight. The avidin-biotinylated horseradish peroxidase complex for a further 30 min. The avidin-biotinylated horseradish peroxidase complex for a further 30 min. Antibody bound to the section was visualized DAB solution, followed by incubation with Bax, Bcl-2, Vimentin, HK2, PKM2, LDHA vimentin after washing the sections with PBS thrice for 5 min, the slices were then treated with avidin-biotin-peroxidase complexes. The information of antibody used in the experiment are shown in Table 2.

### Statistical analysis

All data distribution analyses were evaluated using GraphPad Prism (Version 8.0, GraphPad Software, La Jolla, USA) while evaluating dependent and independent data with normal distribution. Data are presented as mean ± standard deviation. The normal distribution was assessed by Kolmogorov Smirnov test. Significant differences among different treatment groups were analyzed using one-way ANOVA tests followed by Tukey’s test post hoc. *P* < 0.05 was considered statistically significant.

## Results

### Effects of SPM supplement on body weight, testis weight and semen quality

Initially, we divided the animals into four groups and their body weight showed no difference. After 8 weeks of treatment, we observed that SPM treatment mice got a higher weight of body and testis compared to saline-treated diabetic mice. With the increase of SPM concentration, the weight was closer to controls (Table 3). Specifically, sperm quality parameters were also examined, in comparison with control group, diabetic mice got lower weight of body and testis. Sperm count is also rarely, with a plenty of abnormal sperm. While a significant increase was observed in the sperm number of both concentrations of SPM treatment mice. And fewer abnormal sperm and the rates of abnormal sperm were observed compared with saline-treated diabetic mice. It worth noting that the protective effect of 5 mg/kg SPM was closer to that of controls, which is more effective than the concentration of 2.5 mg/kg SPM.

**Table 3:** Effects of SPM treatments on the body weight, testis weight and sperm parameters in diabetic mice

Group	Body weight (g)		Testis weight (mg)	Sperm count (×10^**7**^)	Abnormal sperm number (×10^**7**^)	Abnormal sperm rate (%)
	Pre-induction	After treatment				
**Control**	21.08 ± 0.38	25.36 ± 1.54	173.40 ± 4.65	6.86 ± 0.57	1.81 ± 0.32	25.17 ± 3.91
**DM**	19.52 ± 0.82*	15.40 ± 1.62**	132.40 ± 20.1*	3.60 ± 1.63**	3.00 ± 1.06*	69.09 ± 22.07**
**DM + SPM 2.5 mg/kg**	19.28 ± 0.87**	20.14 ± 1.06^##^	171.20 ± 10.85^#^	5.70 ± 1.36^#^	1.50 ± 0.53^#^	32.15 ± 7.54^##^
**DM + SPM 5 mg/kg**	19.38 ± 0.79**	22.66 ± 1.18^##^	173.53 ± 7.90^#^	6.15 ± 0.63^#^	1.20 ± 0.41^##^	25.17 ± 3.91

*P < 0.05; **P<0.01 vs. Control; ^#^*P* < 0.05; ^##^*P*<0.01 vs. DM

### Effects of SPM supplement on the epididymis, testicular and semen morphology, diameter and area of spermatogenic tubules in diabetic mice

**Fig. 1:**
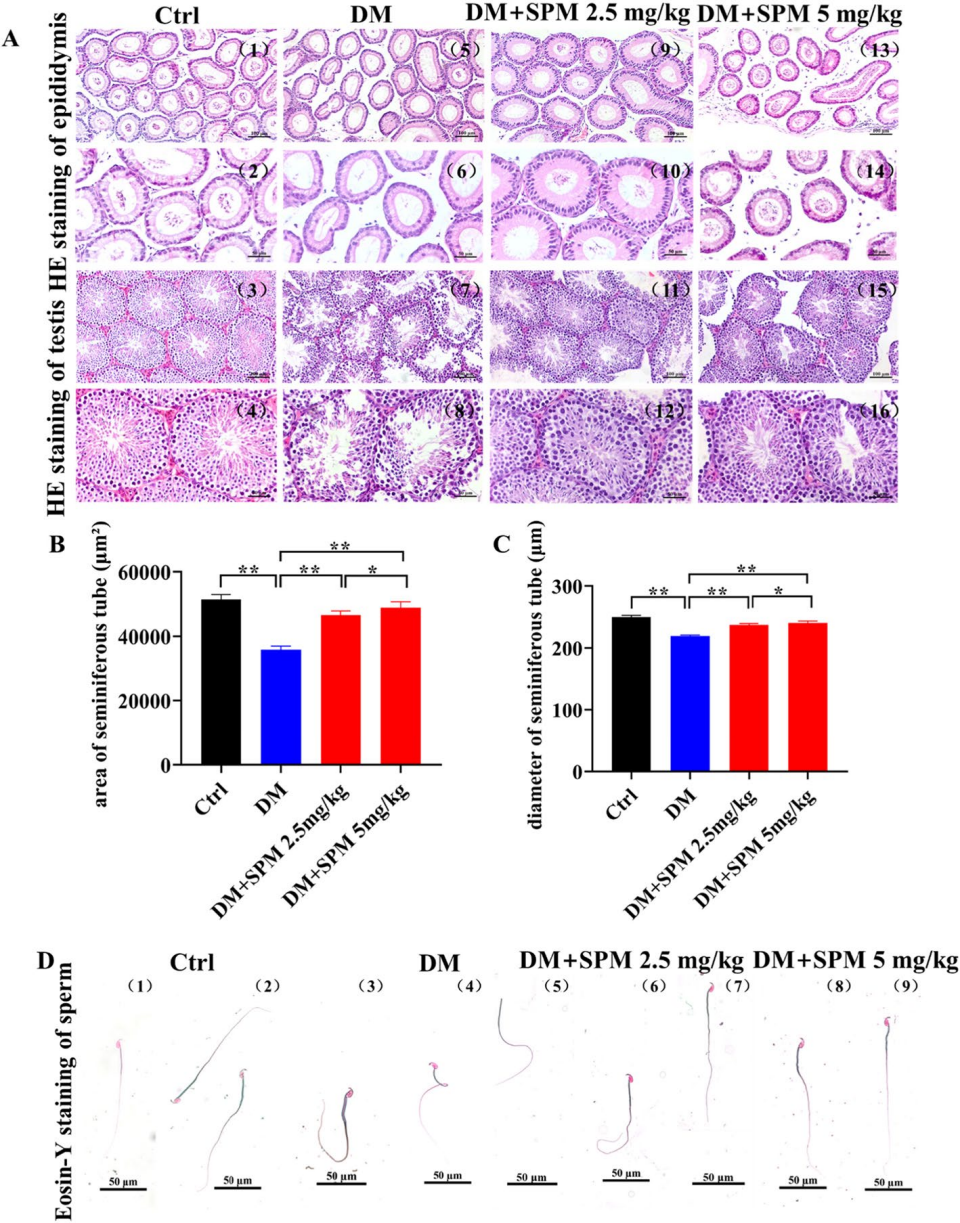
Effects of spermidine on the morphological structure of epididymis and testis in diabetic mice. **A** HE staining of epididymis and testes. **B** The area of seminiferous tube. **C** The diameter of seminiferous tube. **D** The eosin-Y staining of sperms. (1) and (2) are the Ctrl group sperms, (3) (4) and (5) are the DM group sperms, (6) and (7) are the SPM 2.5 mg/kg group sperms, (8) and (9) are the SPM 5 mg/kg group sperms

The epididymis and testicular morphology from various groups was shown in Fig. 1. In the saline-treated diabetic mice, a few sperm can be seen in epididymis (Fig. 1A (5)(6)). However, a large number of sperms were observed in the epididymis of control mice (Fig. 1A (1)(2)) and treated with SPM mice (Fig. 1A (9)(10)), among which mice treated with 5 mg/kg SPM had the highest number of sperm in the epididymis (Fig. 1A (13)(14)). In the saline-treated diabetic mice, the seminiferous tubules were atrophic, with only 1-4 layers of spermatogenic cells in a disordered structure with many vacuoles, the spermatogenic cells were sparsely arranged. In both concentrations of SPM treatments, the seminiferous tubules with regular structure, had multiple layers of densely packed spermatogenic cells. Therefore it preserved the epithelial height (Fig. 1A). The mean area of seminiferous tubules was higher in the control mice (51,357.50 ± 1564.91 μm^2^) than that in the saline-treated diabetic mice (35,871.00 ± 1105.85 μm^2^), 2.5 mg/kg SPM treatment (46,545.41 ± 1274.42 μm^2^) and 5 mg/kg SPM treatment (48,751.93 ± 1877.53 μm^2^; *P* < 0.05 for each group), as shown in Fig. 1B. The diameter of the seminiferous tubular of mice in the control mice (249.85 ± 2.45 μm), 2.5 mg/kg SPM treatment (236.97 ± 2.27 μm) and 5 mg/kg SPM treatment (240.15 ± 3.03 μm) was greater than that in the saline-treated diabetic mice (219.38 ± 1.77 μm; *P* < 0.05) (Fig. 1C). Sperm morphology of the control mice was regular in shape. However, the sperm of the DM group exhibited a disorganized morphology, including the headless, abnormal midpiece, tail curled and deformed, while these were rarely occurred in the control mice sperm. After 2.5 mg/kg SPM treatment, the morphology of part of sperm was improved, but there were still some deformities such as tail curled. While treatment with 5 mg/kg SPM, most sperm had normal morphology (Fig. 1D).

### Effects of SPM supplement on apoptosis in testicular tissues from diabetic mice

**Fig. 2:**
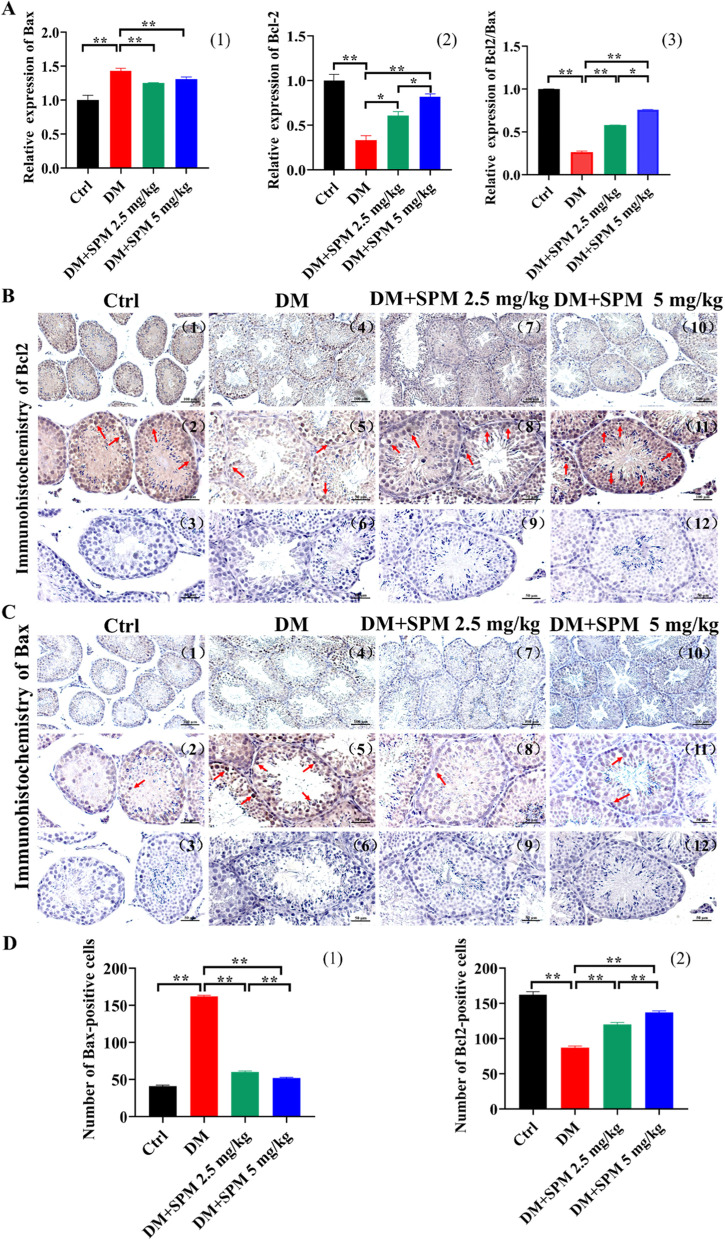
Effects of spermidine on apoptosis of spermatogenic cells in diabetic mics. The red arrows indicate the cells with positive signal. **A** (1) The expression of Bcl-2 in testis detected by qRT-PCR. (2) The expression of Bax in testis detected by qRT-PCR. (3) The expression of Bcl-2/Bax in testis detected by qRT-PCR. **B** The expression of Bcl-2 in spermatogenic tubules was detected by immunohistochemistry. **C** The expression of Bax in spermatogenic tubules was detected by immunohistochemistry. D:(1) Bcl-2 positive cell count in the testis. (2) Bax positive cell count in the testis

We examined the expression of apoptotic factors in testis. The qRT-PCR assay showed that Bcl-2 mRNA expression was downregulated, meanwhile the mRNA expressions of Bax were upregulated in the saline-treated diabetic mice when compared to the control group. And Bcl-2 mRNA expressions were upregulated, with the mRNA expressions of Bax were downregulated in the both concentration of SPM treatment, besides, the ratio of Bcl-2/Bax went down significantly (Fig. 2A). Immunohistochemical results showed the same results (Fig. 2B, C), the red arrows indicate the cells with positive signal. It’s worth noting the function of 5 mg/kg SPM treatment is better than 2.5 mg/kg SPM treatment, in alleviating the diabetes-induced changes in the apoptosis-related mediators (Fig. 2B, C). The number of Bcl2-positive cells was counted under light microscope for statistical analysis. Compared with the diabetic mice, the number of the Bcl2 positive cells in mice treated with 2.5 and 5 mg/kg SPM increased significantly [(87.00 ± 5.05) vs (120.00 ± 2.54), (137.00 ± 2.14), *P* < 0.05], the number of the Bax positive cells in mice treated with 2.5 and 5 mg/kg SPM decreased significantly [(162.00 ± 2.01) vs (60.00 ± 2.34), (52.00 ± 3.10), *P* < 0.05] (Fig. 2D).

### Effects of SPM supplement on SCs number and cytoskeleton changes, and GATA, WT1 expression in testicular tissues from diabetic mice

**Fig. 3:**
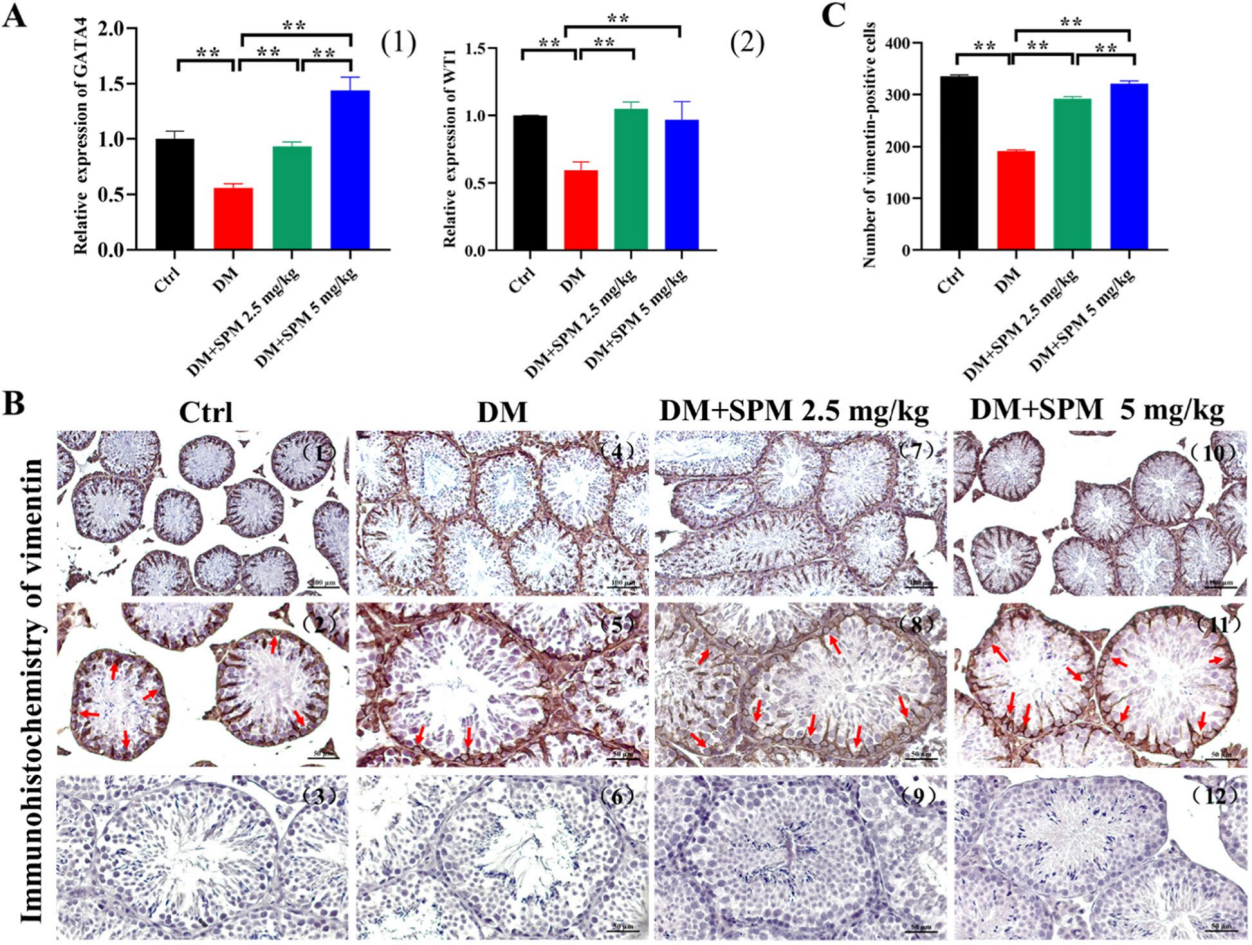
Effects of spermidine on the number and function of testicular Sertoli cells in diabetic mice. The red arrows indicate the cells with positive signal. **A** (1) The expression of GATA4 in testis detected by qRT-PCR. (2) The expression of WT1 in testis detected by qRT-PCR. **B** The expression of Vimentin in spermatogenic tubules was detected by immunohistochemistry. **C** The expression of Vimentin in spermatogenic tubules was detected by immunohistochemistry

**Fig. 4:**
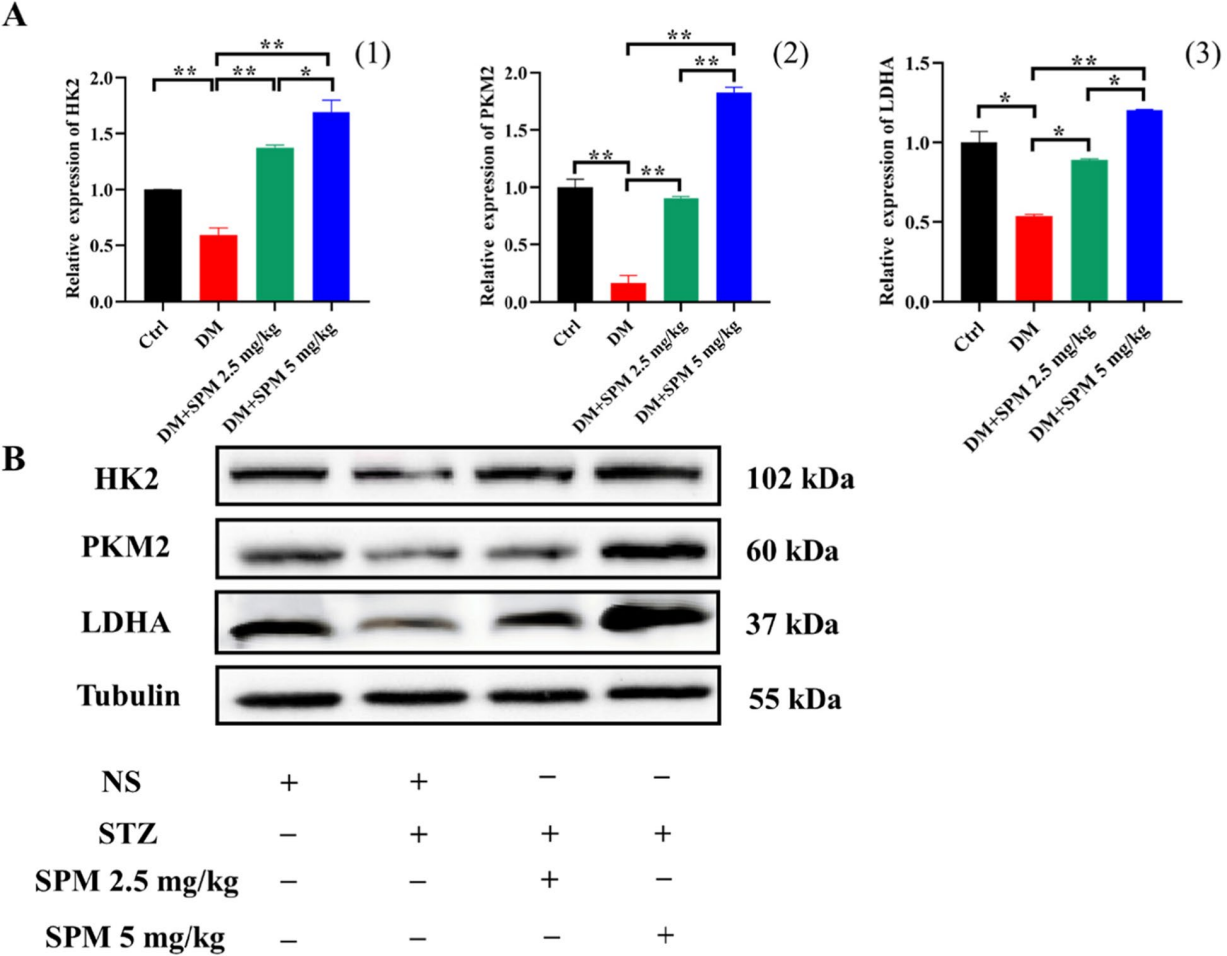
Effects of spermidine glycolytic rate-limiting enzyme gene in diabetic mics testis. **A** (1) The expression of HK2 in testis detected by qRT-PCR. (2) The expression of PKM2 in testis detected by qRT-PCR. (3) The expression of LDHA in testis detected by qRT-PCR. **B** HK2, PKM2, LDHA protein levels in spermatogenic tubules was detected by Western Blot. NS: normal saline

We examined the expression of SCs markers in testis. The mRNA expression of SCs marker gene (GATA, WT1) was downregulated in the testicular tissues of saline-treated DM mice compared to the control group (Fig. 3). Both concentrations of SPM treatments significantly upregulated GATA and WT1 mRNA expression in the testicular tissues compared to saline-treated DM mice (Fig. 3A). Immunohistochemical analysis of Vimentin protein expression in the testicular tissues from the different treatment groups and diabetic mice with saline, which showed a decrease in the number of SCs in the testicular tissues compared to the control group, while the cytoskeleton structure of SCs in SPM 5 group is more in order than SPM 2.5 group (Fig. 3B), The red arrows indicate the cells with positive signal. And compered with diabetic mice, the number of the Vimentin positive cells in mice treated with 2.5 and 5 mg/kg SPM increased significantly [(191.00 ± 1.91) vs (292.00 ± 2.54), (321.00 ± 1.85), *P* < 0.05] (Figs. 3C and 4).

### Effects of SPM supplement on the expression of glycolytic rate-limiting enzyme gene in testicular tissues from diabetic mice

**Fig. 5:**
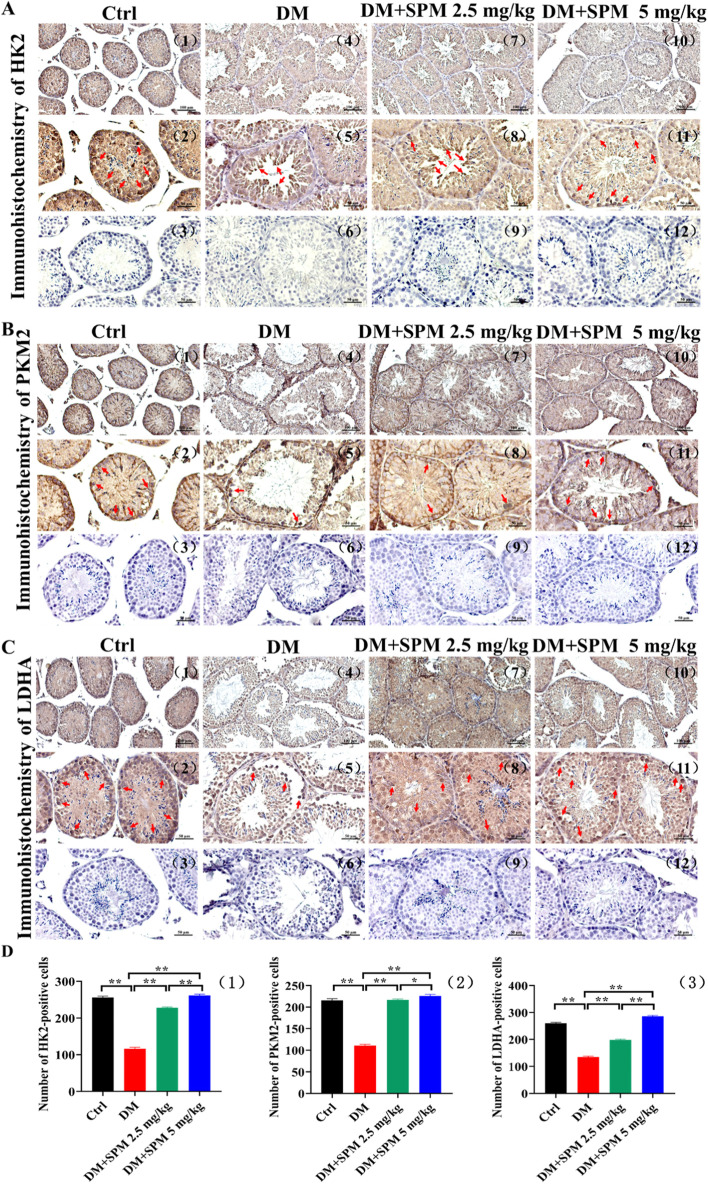
Effects of spermidine glycolytic rate-limiting enzyme gene in diabetic mics testis. The red arrows indicate the cells with positive signal. **A** The expression of HK2 in spermatogenic tubules was detected by immunohistochemistry. **B** The expression of PKM2 in spermatogenic tubules was detected by immunohistochemistry. **C** The expression of LDHA in spermatogenic tubules was detected by immunohistochemistry. **D** (1) HK2 positive cell count in the testis. (2) PKM2 positive cell count in the testis. (3) LDHA positive cell count in the testis

We examined the expression of glycolytic rate-limiting enzyme gene in the testis. In the qRT-PCR assay, the mRNA expression of HK2, PKM2 and LDHA were significantly decreased in diabetic mice with saline, which was almost improved in the SPM 2.5 mg/kg group and SPM 5 mg/kg group (*P* < 0.05; Fig. 4A), which protein levels in the same trend by Western blot (Fig. 4B). Immunohistochemical analysis confirmed that the expression of glycolytic rate-limiting enzyme gene (HK2, PKM2, LDHA)-positive cells in STZ-treated groups was significantly decreased compared with the control groups (*P* < 0.05; Fig. 5A, B, C, D), The red arrows indicate the cells with positive signal. Consistently, SPM treatments in both concentrations increased the number of HK2, PKM2 and LDHA-positive cells in the testicular tissues of diabetic mice when compared to treatment with saline (*P* < 0.05; Fig. 5A, B, C, D).

## Discussion

It was reported that the proportion of infertility couples accounts for about 1/10 of the total population worldwide, and half the causes lie in male [19]. The onset of diabetes mellitus (DM) is becoming younger and younger, which leads to sexual dysfunction, abnormal testicular structure and spermatogenic dysfunction, which is one of the important reasons for male infertility [20]. Overall, hyperglycemia is an important marker of type 1 diabetes mellitus (T1DM), numerous studies have shown that hyperglycemia give rise to a decrease in male fertility by damaging the reproductive endocrine of the hypothalamic-pituitary-gonadal axis [21], which could induce abnormal spermatogenesis. Moreover, hyperglycemia in testis aggravated the morphological and functional changes of testis and epididymis, impaired semen quality as well as dwindled sperm count, sperm motility and semen volume [1, 22]. In the previous studies, using a single intraperitoneal injection of 120 mg/kg STZ [23], the STZ-induced diabetic mice was constructed successfully, along with an increase in blood sugar, the body weight and testicles weight of DM mice was significantly reduced [24]. Meanwhile, serious histological damage, including reduced cross-sectional area of spermatogenic tubules and reduced diameter and morphological destruction was caused [25]. What’s more, reduced spermatogenic cells and sperm count, and increased rate of malformed sperms are detected in DM mice [26]. In this study, our results were consistent with all of the above literatures, which makes meaningful by exploring the spermatogenic dysfunction in diabetic males forward.

Spermidine (SPM) is a sufficient natural polyamine, which contained in all organisms from bacteria to human and presented in diets, such as nuts, beans and so on. Studies have shown that SPM has a variety of pharmacological functions such as anti-inflammatory, anti-oxidation, promoting autophagy and inhibiting lipid formation [9, 10, 27]. Excitingly, SPM has a protective effect on the DM, exogenous SPM supplementation could alleviate myocardial fibrosis in diabetic cardiomyopathy [12]. Besides, SPM also could attenuate hemoglobin glycation and lipid peroxidation in diabetes rats [13, 28]. Meanwhile, It has been found that SPM plays pleiotropic functions, such as it guarantees sperm to perform its acrosomal reaction at the right time [29], improves sperm motility [30], increases the rate of normal sperm morphology and enhances superoxide dismutase activity [31]. Recent studies have shown that intaking exogenous spermine could rescue triptolide-induced testicular dysfunction, by increasing the expression of genes related to spermatogenic events and increasing the reduced number of offspring [32]. Addition of L-arginine and polyamines to human sperm cells from diabetic asthenozoospermic patients played a beneficial role on sperm motility [33]. In the present study, the decreased in the number of abnormal sperm, significantly increased in the weight of testis and epididymis as well as sperm count were detected in DM mice after SPM supplementation, which indicated a good improvement of SPM on the spermatogenesis dysfunction in DM mice. Furthermore, more and more literatures have proved that in the testes with hyperglycemia, the expression profiles of apoptosis related proteins Bax and the Bax/Bcl-2 ratio were upregulated, as well Bcl-2 was downregulated, which was accompanied with the increasing number of apoptotic spermatogenic cells [34, 35]. While SPM have been clarified that it could abrogate the apoptosis of spermatogenic cells by reducing apoptosis independent of autophagy, upregulating Bcl-2 and decreasing Bax expression [36, 37]. Our findings revealed that in DM mice, SPM played an important role in preventing apoptosis through up-regulating Bcl-2 and down-regulating Bax in the testes. Furthermore, this study explained that SPM could significantly reduce spermatogonia dysfunction in male diabetes by against apoptosis mediated in spermatogenic cells, while it has not been reported in previous literature.

Interestingly, SCs are the only somatic cells in the seminiferous tubules that can secrete a variety of growth factors and play a key role in spermatogenesis [38]. Nevertheless, SCs are often the target cells of external toxic substances, which have toxic actions on male reproductive function by damaging the structure and function of SCs [39]. Patients with DM are often accompanied by low levels of androgen and reduction of inhibin B and AR expression, which suggested SCs function is destroyed [40]. High glucose level in DM prevented the maturation of SCs in cultured testicular tissue, resulting in a decreased number of SCs and a significant decrease in the area of spermatogenic tubules [41]. Vimentin, WT1 and GATA are marker genes of SCs, which are stably expressed in the nucleus of testicular SCs. Especially, the expression of Vimentin is associated with epithelial structural integrity [42], which forms the skeletal structure of the SCs [43]. In the testis of STZ-induced diabetic mice, the expression of Vimentin was down-regulated, the cytoskeleton structure of SCs was destroyed, and the cell function decreased as well [44]. Results in the present study was consistent with previous studies in our DM mice, but the expression of Vimentin was upregulated after SPM supplement, which suggesting that SPM plays a vital role in maintaining the regular cytoskeleton of seminiferous tubule in diabetic mice. In advance of this study, the expressions levels of WTI and GATA in testis were decreased in diabetic mice, which manifesting the decline in the number and function of SCs [22, 45]. In our study, we also verified a significant decrease in the expression levels of WT1 and GATA in diabetic testis, which was eliminated by SPM supplement in DM mice. Summarily, DM impairs the cytoskeleton structure and function of SCs, SPM can be used to improve diabetes by reversing the morphology, structure and function of SCs.

In general, testis has been reported to be a naturally oxygen-deprived organ, energy produced by glycolysis of SCs is an important metabolic control point for spermatogenesis, as well SCs could quickly provide adenosine triphosphate and lactate to meet the needs of rapidly proliferating spermatogenic cells for energy and substances [46, 47]. The regulatory mechanism during the process of transporting glucose to SCs, LDHA catalyzing pyruvate into lactate, as well the transporting lactate to spermatogenic cells plays an important role in spermatogenesis [47, 48]. Previous studies have demonstrated that the LDHA is reduced in testes from T1DM patients [49], similarly, the expression of key glycolytic enzymes HK2, PKM2 and LDHA in the testis of diabetic mice are significantly reduced, which leads to glycogen accumulation, cut down of lactate production of SCs, eventually disorders the energy metabolism of spermatogenic cells and induces a significant cell apoptosis [50, 51]. Pioglitazone as a hypoglycemic drug that functions by enhancing insulin sensitivity in the target tissue, which can stimulate the glucose consumption in human SCs to more than double, and increase the glycolysis rate and lactate production, thus, the better medicine for maintaining and improving the spermatogenic events caused by DM [52]. Similarly, lipoic acid has been reported to enhance the absorption of glucose into cells and improve islet function and glucose metabolism, its supplementation could inhibit the apoptosis of SCs and increase the ability of ATP production, which improved sperm motility and concentration in DM rats. When SCs are exposed to pharmacological doses of L-theanine (50 μM), it could promote cell proliferation and glycolysis rate, maintain energy requirement for cell component synthesis, and support to build new cells [53]. Meanwhile, SPM metabolism is sensitive to glycolysis impairment in tumor cells [54], mechanistically, when SPM biosynthesis is impaired, the reduced aerobic glycolysis could inhibit cell proliferation and promote inflammation [55]. Our experimental results have proved that DM could down-regulated the expressions of HK2, PKM2 and LDHA in the testis of DM mice, while SPM supplementation increased the mRNA and protein expressions of HK2, PKM2 and LDHA in the testis of DM mice. By the same time, when DM patients take a combination of insulin and melatonin for the therapy, which could better maintain the protein levels of glycolysis-related enzymes at normal levels and improve the dyszoospermia [56]. However, the findings of this study are still in the preliminary phases, the latent molecular mechanisms, such as what factors mediate the change of glycolysis rate-limiting enzyme gene in diabetes mice with the SPM supplement, are still vague and require further investigation.

**Fig. 6:**
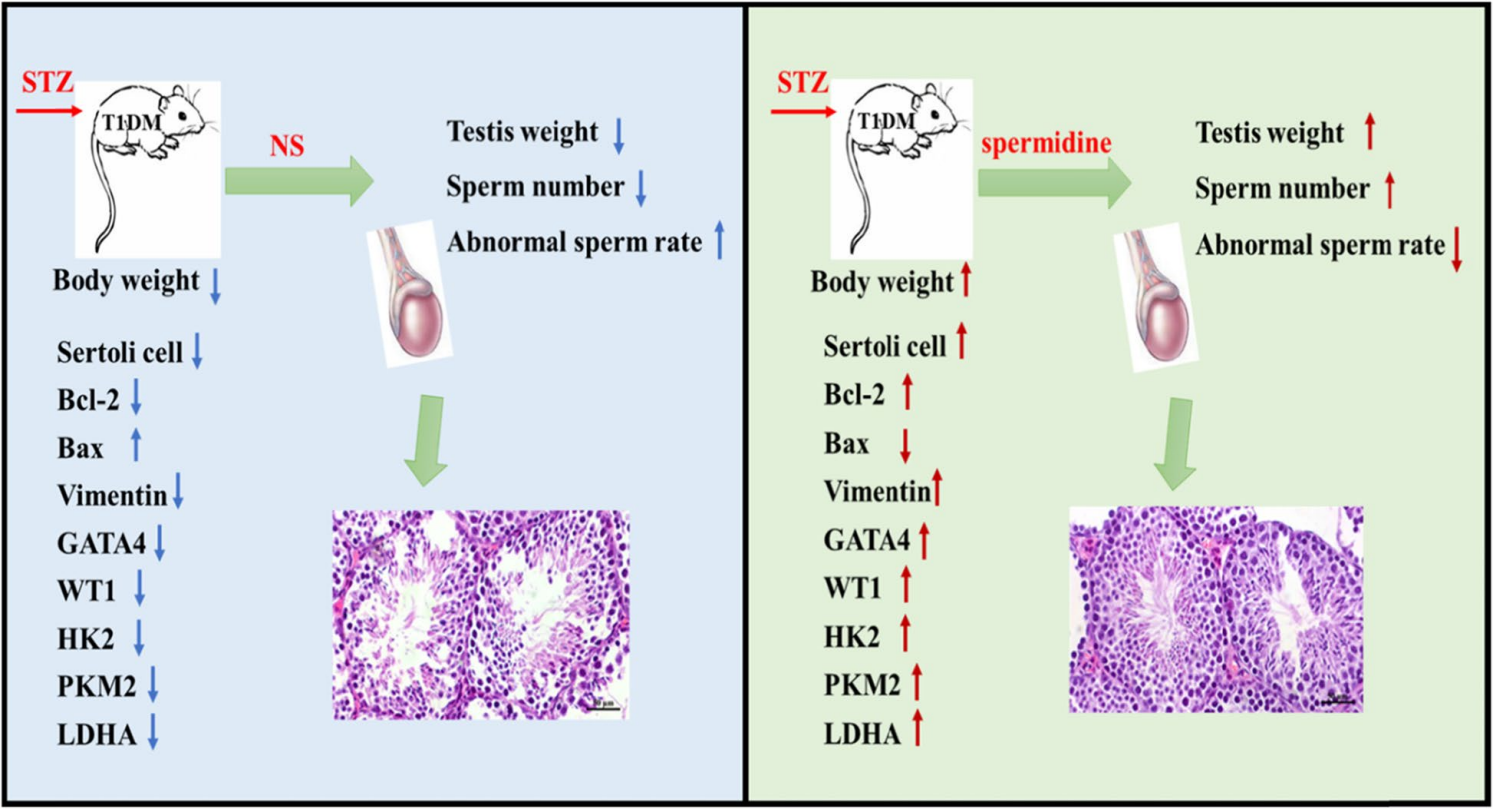
In vivo supplementation of the spermidine (SPM) effectively protective DM-induced male reproductive damage by improving the pathological structure of testis, inhibiting apoptosis of spermatogenic cells and promoting proliferation. Eventually, SPM could ameliorate the structure and function of SCs in DM mice, increase the expression of glycolytic rate-limiting enzyme

In conclusion, our results manifested that the protective effects of SPM on the DM-induced male reproductive damage in mice by improving the pathological structure of testis, inhibiting apoptosis of spermatogenic cells and promoting proliferation. Eventually, mechanistic results showed that SPM ameliorated the structure and function of SCs in DM mice, increased the expression of glycolytic rate-limiting enzyme, and that the upregulated HK2, PKM2, LDHA expression may have a positive effect on the dyszoospermia in DM mice (Fig. 6).

## Data Availability

Not applicable.

## Abbreviations

SPM: Spermidine
DM: Diabetes mellitus
T1DM: Type 1 diabetes mellitus
STZ: Streptozotocin
SCs: Sertoli cells
WT1: Wilmstumor1
HK2: Hexokinase 2
PKM2: Pyruvate kinase M2
LDHA: Lactate dehydrogenase A
